# Right Atrial Dose Is Associated with Worse Outcome in Patients Undergoing Definitive Stereotactic Body Radiation Therapy for Central Lung Tumors

**DOI:** 10.3390/cancers14061391

**Published:** 2022-03-09

**Authors:** Mark Farrugia, Han Yu, Sung Jun Ma, Austin J. Iovoli, Saraswati Pokharel, Umesh C. Sharma, Simon Fung-Kee-Fung, Nadia Malik, Anurag K. Singh, Harish Malhotra

**Affiliations:** 1 Roswell Park Comprehensive Cancer Center, Department of Radiation Medicine, Buffalo, NY 14203, USA; mark.farrugia@roswellpark.org (M.F.); sungjun.ma@roswellpark.org (S.J.M.); austin.iovoli@roswellpark.org (A.J.I.); simon.fung-kee-fung@roswellpark.org (S.F.-K.-F.); nadia.malik@roswellpark.org (N.M.); harish.malhotra@roswellpark.org (H.M.); 2 Roswell Park Comprehensive Cancer Center, Department of Biostatistics & Bioinformatics, Buffalo, NY 14203, USA; han.yu@roswellpark.org; 3 Roswell Park Comprehensive Cancer Center, Department of Pathology & Laboratory Medicine, Buffalo, NY 14203, USA; saraswati.pokharel@roswellpark.org; 4 Department of Medicine, Jacobs School of Medicine & Biomedical Sciences, University at Buffalo, Buffalo, NY 14203, USA; sharmau@buffalo.edu

**Keywords:** SBRT, lung cancer, survival, dose constraints, heart, cardiac substructures

## Abstract

**Simple Summary:**

The clinical consequences of irradiating the cardiac substructures during stereotactic body radiation therapy (SBRT) remains unclear. We evaluated 83 lung cancer patients who underwent SBRT for early stage lung cancer. Using specialized software, we generated structures for fourteen cardiac substructures and evaluated radiation dose parameters for each. Among these parameters, the dose to 45% (D45%) of either the right atria or ventricle was associated with worse non-cancer associated survival with an identified cutoff value of 890 cGy and 564 cGy for each, respectively. Via these cutoffs, the D45% to the right atria, not the right ventricle, was associated with worse non-cancer associated and overall survival. Based on these findings, reducing the dose to the right atria during SBRT may improve patient outcomes in at risk patients.

**Abstract:**

The consequence of cardiac substructure irradiation in patients receiving stereotactic body radiation therapy (SBRT) is not well characterized. We reviewed the charts of patients with central lung tumors managed by definitive SBRT from June 2010–April 2019. All patients were treated with five fractions, typically either 5000 cGy (44.6%) or 5500 cGy (42.2%). Via a multi-patient atlas, fourteen cardiac substructures were autosegmented, manually reviewed and analyzed using dosimetric parameters. A total of 83 patients were included with a median follow up of 33.4 months. Univariate Cox regression analysis identified a D45% dose to the right atria and ventricle for further study. Sequential log-rank testing evaluating an association between non-cancer associated survival and D45% dose to the right atria or ventricle and association was employed, identifying candidate cutoff values of 890.3 cGy and 564.4 cGy, respectively. Kaplan–Meier analysis using the reported cutoff values found the D45% right atria constraint to be significantly associated with non-cancer associated (*p* ≤ 0.001) and overall survival (*p* ≤ 0.001) but not the right ventricle constraint. Within a multivariate model, the proposed right atria D45% cutoff remained significantly correlated with non-cancer associated survival (Hazard’s Ratio (HR) ≤ 8.5, 95% confidence interval (CI) 1.1–64.5, *p* ≤ 0.04) and OS (HR ≤ 6.1, 95% CI 1.0–36.8, *p* ≤ 0.04). In conclusion, a dose to D45% of the right atria significantly correlated with outcome and the candidate constraint of 890 cGy stratified non-cancer associated and OS. The inclusion of these findings with previously characterized relationships between proximal airway constraints and survival enhances our understanding of why centrally located tumors are high risk and potentially identifies key constraints in organ at risk prioritization.

## 1. Introduction

Lung cancer leads the world in cancer-related mortality with an estimated 1.8 million deaths in 2020 alone [1]. While surgery is the standard of care in patients who are eligible, radiotherapy remains one of the major treatment modalities for either early stage or locally advanced disease [2]. Despite its efficacy, there has been a recent focus on cardiac toxicity in patients receiving definitive radiation treatment for non-small cell lung cancer (NSCLC) [3,4,5,6,7,8,9,10].

In RTOG 0617, efforts to escalate radiation doses within locally advanced NSCLC were unsuccessful; however, important relationships between heart dosimetry and outcome were uncovered [8,11]. The impact of radiation doses on cardiac substructures in patients treated with conventional fractionation for stage III NSCLC has been further explored [7,8,9,10].

The dose relationship between cardiac substructures and patient outcome following SBRT is less well established [4,6]. Previously, we demonstrated that exceeding 18 Gy to 4 ccs to either the proximal bronchus or trachea was associated with worse outcomes in patients with NSCLC treated with five-fraction stereotactic body radiation therapy (SBRT) for central and ultracentral lung tumors [12]. Doses to cardiac substructures were lacking in that analysis.

To address this knowledge gap, we employed a previously described multi-patient atlas to autosegment fourteen cardiac substructures in patients with central or ultracentral lung tumors undergoing definitive SBRT [13]. These dosimetric findings were correlated with survival endpoints to identify potential constraints for future studies. 

## 2. Methods

### 2.1. Patient Population

The patient cohort was derived from a collection of 438 primary NSCLC patients who underwent SBRT for thoracic tumors from February 2007 to April 2019 [14]. To investigate the impact of heart irradiation during SBRT, we restricted our analyses to those with central lung tumors who were treated with a five-fraction regimen for a total of 83 patients. Data were collected under approval from the institutional review board at Roswell Park Comprehensive Cancer Center (EDR-171710).

### 2.2. Clinical Evaluation and Follow-Up

The clinical work up and evaluation to determine eligibility for SBRT was previously described [12,14]. In short, patients who either were not surgical candidates, unwilling to have a pneumonectomy or refused surgical resection were evaluated for SBRT. All patients were cN0 as determined by positron emission tomography with diagnostic computed tomography (PET/CT) imaging and/or endoscopic nodal sampling. Follow up schedule included a diagnostic CT chest 3 months post-treatment followed by repeat imaging every 3–6 months up to a year [12,14]. Following a year post-treatment, chest CTs were performed every 6 months. PET/CT imaging was ordered for concerning findings with biopsies as necessary. Progression was defined as clear growth of known lesions or new lesions on imaging with or without pathologic confirmation.

### 2.3. Patient Data

Pertinent clinicopathologic data were obtained using chart review. The staging was performed via the American Joint Commission on Cancer 8th edition and definitions for clinical conditions, such as diabetes mellitus, history of heart disease, and previous lung cancer were previously described [12,14]. Heart disease was defined as a history of congestive heart failure (CHF), a history of coronary artery disease (CAD), or a history of myocardial infarction (MI). Performance status was defined by Karnofsky Performance Status (KPS). Central tumors were defined as 2 cm within the proximal airway, mediastinum, great vessels, or spinal cord whereas ultracentral tumors were directly abutting any of the above structures [12].

### 2.4. SBRT

All patients underwent five-fraction SBRT. Patient setup, motion management, delivery techniques, and dose prescription were previously described [12,14,15].

### 2.5. Heart Substructures

Structures were defined per the atlas described by Feng et al. [16]. The cardiac substructures were generated using MIM (v 6.9.6, Beachwood, OH, USA) which applied a multi-patient atlas for autosegmentation [13]. Each structure was manually reviewed by a senior radiation oncology resident (MF) and edited if necessary. In general, the heart chambers and large vessels required modest changes, whereas the heart valves and coronary arteries typically required manual definition [13].

### 2.6. Dosimetric Analysis

Eclipse (Varian Medical Systems, Palo Alto, CA, USA) was used for the generation and evaluation of radiation treatment plans. Proximal airway constraints were previously characterized and obtained per RTOG 0813 [12,17]. Dose parameters were reported using the terminology D (volume or percent of the structure, e.g., D45% corresponds to the minimum dose that 45% of the structure received). The D45% parameter for heart chambers was obtained from Thor et al. [8]. For large vessels, D10 cc and maximum doses were evaluated per the SUNSET trial with the exception that D2 cc was employed for the superior vena cava (SVC) given the typical SVC volume was between 10–15 cc [13,18]. Dose to volume metrics were also collected for other structures using 2 ccs for the heart chambers whereas 0.1 ccs were used for small structures including the heart valves and coronary arteries. Lastly, the mean dose to the coronary arteries and heart valves were also recorded. 

### 2.7. Statistics

Time to progression was defined as the date of treatment to date of documented progression as determined by imaging or biopsy. Patients who died without a history of progression were censored. Similarly, non-cancer associated survival was recorded from the date of treatment to the date of death in patients with no history of progression. Those who died with a history of progression were censored. Overall survival (OS) was defined as the date of treatment to the date of death due to any cause. For all relevant endpoints, patients who were lost to follow-up prior to an event were censored. To evaluate potential relationships between cardiac substructure dose and outcome, univariate Cox regression was performed for each recorded dosimetric parameter and non-cancer associated survival. Variables with *p*-values < 0.05 were selected for further analysis. To identify cutoff values that could serve as dose constraints, we dichotomized the group by each observed value between the 20th and 95th percentile of the selected variable and compared the risk of non-cancer deaths between two groups by log-rank tests. The cutoff of the corresponding variable was selected to minimize the *p*-value. Note that multiplicity is not a concern here because this partitioning is conditional on an overall significant association between the selected parameter and the survival outcome. The corresponding cutoff values were then evaluated for associations between relevant outcomes including time to progression, non-cancer associated survival, and OS using Kaplan–Meier survival estimation with log-rank test. To support these findings, competing risk and cumulative incidence analysis was also performed with respect to time to progression and non-cancer associated survival. Lastly, these values were then incorporated into a multivariate Cox regression model including several other clinicopathologic variables which were previously shown to correlate with outcome in this data set [12]. All *p*-values were two-sided. Variables with *p* < 0.05 were considered significant. Statistical analyses were performed using R v 4.0.2.

## 3. Results

A total of 83 patients were included (Table 1). The median age was 73.1 years (interquartile range [IQR] 66.6–78.4 years) with a median follow up of 33.4 (IQR 14.9–52.4) months. The majority were KPS 80–100 (71.1%). All patients were treated with five fractions, typically either 5000 cGy (44.6%) or 5500 cGy (42.2%). Motion management was utilized in all patients, most commonly by respiratory gating (80.7%). The cohort was near evenly split between central (48.2%) and ultracentral (51.8%) tumor locations. Recurrences were documents in 30 (36.1%) of patients. Regarding patients who relapsed, 33% went on to receive systemic treatment whereas 33% underwent local therapies often with palliative intent. See Supplementary Table S1 for further details on those with heart disease.

Radiation dose metrics to cardiac substructures are reported in Table 2. Given that the doses to each respective structure are tumor location dependent, there was a large range in reported values to each structure.

To screen for a potential relationship between radiation dose and outcome, we performed univariate Cox regression for each recorded parameter and non-cancer associated survival, identifying D45% to the right atria (*p* ≤ 0.021) or right ventricle (*p* ≤ 0.012) for further study (Table 3).

**Figure 1:**
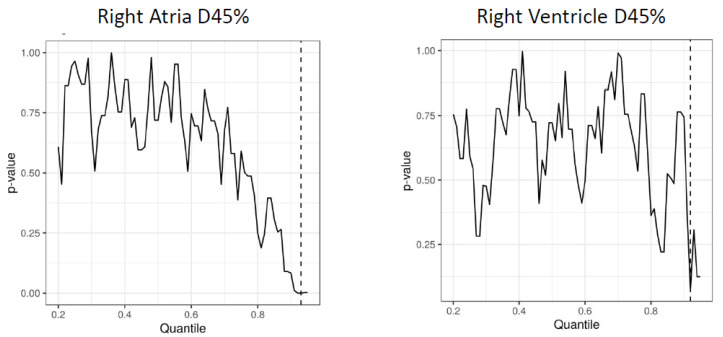
Dichotomization of right atria (**left**) and ventricle (**right**) D45% by observed values between the 20th and 95th percentile and corresponding risk of non-cancer death by log-rank tests. Lowest observed *p*-value denotes by vertical dashed line.

Univariate screen by OS yielded similar results (Supplementary Table S2). To identify candidate radiation dose cutoff values, log-rank analysis was performed for non-cancer associated survival and values between the 20th and 95th percentile of each of the selected dosimetric parameters. Via this method, we identified the 92nd percentile (890.3 cGy) and the 93rd percentile (564.4 cGy) for D45% to the right atria and ventricle, respectively (Figure 1).

**Figure 2:**
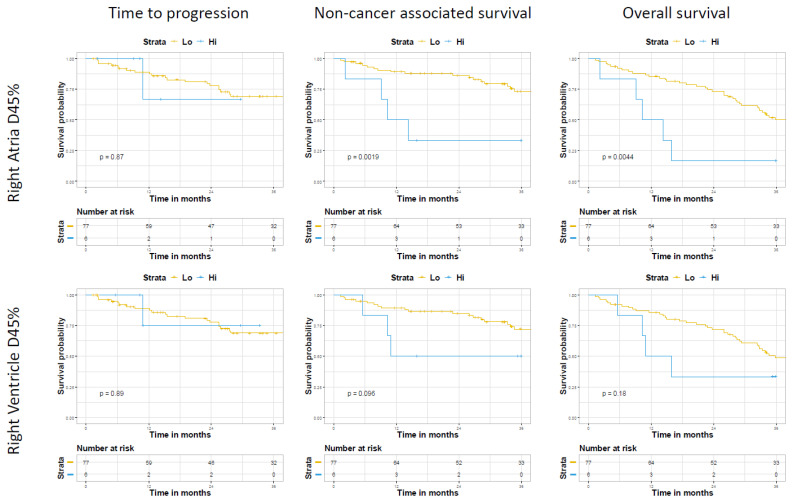
Associations between selected the cutoff values for right atria (**top**) and ventricle (**bottom**) D45% and relevant outcomes by Kaplan Meier.

Kaplan–Meier analysis using the reported cutoff values found the D45% right atria constraint to be significantly associated with non-cancer associated survival (*p* ≤ 0.001) and OS (*p* ≤ 0.001) but not the right ventricle constraint (Figure 2). Neither parameter was associated with time to progression (Figure 2). Competing risk analysis did not reveal a significant relationship between non-cancer associated survival and either constraint (Right atria D45% (Hazard’s Ratio (HR) ≤ 5.8, 95% confidence interval [CI] 0.14–245, *p* ≤ 0.36); right ventricle D45% (HR 0.15, 95% CI 0.0009–23.2, *p* ≤ 0.46).

To further evaluate these findings, we performed multivariate Cox regression incorporating several variables previously shown to be significantly associated with outcome in this cohort (Table 4). Within this model, right atria D45% remained significantly correlated with non-cancer associated survival (HR ≤ 8.0, 95% CI 1.0–62.5, *p* ≤ 0.048) and OS (HR ≤ 7.4, 95% CI 1.2–45.7, *p* ≤ 0.029). Similar results were obtained when ultracentral tumor location was substituted for D4 cc to the bronchus and trachea (Supplementary Table S3).

## 4. Discussion

Radiation doses to the cardiac substructures with five fraction SBRT for central lung tumors ranged widely from nearly zero to near the prescription dose. Multivariate analysis revealed the D45% right atria constraint to be associated with non-cancer associated and overall survival. Sequential log-rank testing identified a candidate cutoff value of 890.3 cGy.

Radiation-association cardiotoxicity remains a major concern for lung cancer treatments. RTOG 0617, a randomized phase III clinical trial, showed a survival detriment with dose escalation in locally advanced NSCLC [11]. In this trial utilizing conventionally fractionated radiation, the volume of the heart receiving 5 Gy (V5) was associated with reduced OS [11]. Other groups failed to validate heart V5 in independent cohorts but offered several alternative candidate constraints implicated in survival [7,19,20]. Speirs et al. found a heart V50 of 25% to significantly stratify 1-year OS (70.2% vs. 46.8%) [19].

Some studies support cardiac subsite specific versus whole heart constraints. McWilliam et al. found no significant correlations between mean heart dose (MHD), V5 and V30 and outcome, however, permutation testing found excess doses to the base of the heart to be associated with increased mortality [7]. Vivekanandan et al. found radiation doses to the walls of the left and right atria to be correlated with all-cause death rate. Ref. [10] Thor et al. reported an averaged model including atria D45%, mean dose of the hottest (MOH) 55% to the pericardium, a MOH5% to the ventricles, and lung mean dose which had excellent performance in predicting OS within the RTOG 0617 dataset [8].

In contrast to these reports on conventionally fractionated radiation therapy, studies of the impact of cardiac substructure doses on survival following SBRT are limited. As previously published, there was no association between heart D_max_, D10 and D15 cc doses, and heart D45% with non-cancer associated survival in the current cohort [12].

Stam et al. reported the D_max_ to the left atrium, D90% to the superior vena cava, and doses to the upper region (left and right atrium, and vessels) to be associated with worse non-cancer associated death [6]. While right atrial parameters were significantly correlated with outcome in the current study, doses to the left atrium and vessels were not. Three notable differences between the studies may account for these discrepancies. First, nearly 95% of their patients had peripheral tumors. Second, they were mostly treated with 54 Gy in three fractions [6]. Third, Stam et al. employed deformable registration to delineate the cardiac substructures without manual review. In our experience, autosegmented structures routinely require some editing [13].

Reshko et al. also examined the consequence of heart substructure irradiation during SBRT, finding only the mean heart dose and none of the substructure specific parameters to correlate with outcome [21]. Within this report, nearly half of the patients had central tumors and cardiac substructures were defined by the same atlas utilized in the current study [16,21]. Despite these commonalities, Reshko et al. focused exclusively on mean dose and D_0.03cc_ as dosimetric parameters for each substructure which may account for the discordant results.

Despite evidence suggesting cardiac toxicity can impact survival in patients undergoing thoracic radiation, currently there are no clear recommendations for cardiac surveillance in these patients. At risk patients could undergo comprehensive cardiac echocardiography with a specific assessment of right atrial morphology and function. Furthermore, telemonitoring approaches can be utilized for the early detection of subtle (occult) cardiac arrhythmias—thus allowing rationale for pharmaceutical interventions (ACEi/ARBs, rate-control agents) which may attenuate RT-related cardiotoxicity. In addition, we are in the process of testing this hypothesis in a pre-clinical (rodent) model of cardiac irradiation, which will help elucidate the mechanism of RT-related damage in this scenario. The cardiovascular aspects of our current research were obtained in collaboration with one of our collaborators (UCS), who has specialized training and experience in advanced cardiac imaging. 

In this study, in multivariate analysis, D45% to the right atria was the only significant predictor of non-cancer associated or overall survival with radiation doses to the cardiac substructures. Despite these results, we would not conclude that radiation doses to other cardiac substructures are inconsequential. Univariate Cox regression was performed on continuous dosimetric values as a screen to identify candidate parameters but it is possible that dichotomization of these variables would have yielded significant findings. Additionally, due to limited sample size, in depth dose modeling could not be performed, and therefore, there could be other relevant dosimetric parameters that were not investigated in this study (e.g., MOH5%, D90%). Lastly, radiation-induced damage of certain structures may occur over longer timeframes than the expected survival within this older and surgically ineligible cohort. Therefore, caution should be exercised when applying these findings to younger and healthier patients.

Other limitations include: (1) contouring was performed on a CT average scan of select respiratory phases without IV contrast. Therefore, the definition of certain structures, such as the heart valves and distal portions of the coronary arteries was challenging. (2) Analyses were restricted to patients with either central or ultracentral tumors to enrich for patients at highest risk for radiation induced cardiac damage. Such patients account for approximately 20% of our patient population [14]. Therefore, it is unclear how applicable these findings are in the 80% of patients who present with peripheral tumors [14]. Investigation on the impact of right atrial doses in peripherally located lung tumors is currently underway. (3) Only a small number of patients (*n* ≤ 6) exceeded 890 cGy D45% to the right atria. (4) Competing risk analysis failed to show a significant relationship between the right atria D45% cutoff and non-cancer associated survival. Consequently, verification in an independent cohort is needed. (5) The dose exposure to the cardiac substructures is expectedly quite heterogeneous and together with the limited study size, this can prohibit alternative statistical approaches to characterizing dose relationships with the outcome (e.g., ROC-based methods). (6) The mechanism between right atrial doses and worse survival was not explored in this study. It is unclear whether right atria irradiation damages the conduction system or produces structural anomalies, and the cause of death could not be determined for most patients. We plan to investigate this knowledge gap in future translational investigations.

In conclusion, doses to D45% of the right atria were significantly correlated with outcome and the candidate constraint of 890 cGy significantly stratified non-cancer associated and OS. The inclusion of these findings with previously characterized relationships between proximal airway constraints and survival enhances our understanding of why centrally located tumors are high risk and potentially identifies key constraints in organs at risk prioritization.

## Figures and Tables

**Table 1:** Patient demographics.

		Median (IQR)	*n*	%
Age (Years)		73.1 (66.6–78.4)		
Sex	Male		39	47.0%
	Female		44	53.0%
Karnofsky Performance Status	80–100		59	71.1%
	<80		24	28.9%
Tumor size	<2 cm		57	68.7%
	2–5 cm		26	31.3%
Ultracentral	No		40	48.2%
	Yes		43	51.8%
Laterality	Left		44	53.0%
	Right		39	47.0%
Nodal Sampling	No		45	54.2%
	Yes		38	45.8%
Tobacco pack years	<30 pack years		23	27.7%
	30+ pack years		60	72.3%
Diabetes	No		65	78.3%
	Yes		18	21.7%
Heart disease	No		49	59.0%
	Yes		34	41.0%
Prior treated lung cancer	No		60	72.3%
	Yes		23	27.7%
Dose (5 fractions)	5000		37	44.6%
	5250		7	8.4%
	5500		35	42.2%
	5750		2	2.4%
	6000		2	2.4%
Technique	3DCRT		49	59.0%
	VMAT		34	41.0%
Tumor motion Management	Respiratory Gating		67	80.7%
	Abdominal Compression		16	19.3%
GTV volume (cm^3^)		9.1 (4.7–23.1)		
PTV volume (cm^3^)		31.0 (18.0–53.3)		
Relapse	No		53	63.9%
	Yes		30	36.1%
Vital Status	Alive		27	32.5%
	Dead		56	67.5%
Follow-up (months)		33.4 (14.9–52.4)		

**Table 2:** Radiation dose to cardiac substructures.

	Median (cGy)	25th Percentile (cGy)	75th Percentile (cGy)	Minimum (cGy)	Maximum (cGy)
**Large Vessels**					
Superior Vena Cava D2 cc	600.13	144.27	1187.38	14.94	4330.72
Superior Vena Cava Dmax	974.99	427.41	1823.43	11.82	6755.42
Pulmonary Artery D10 cc	554.85	128.58	986.96	12.45	3018.34
Pulmonary Artery Dmax	1393.26	613.5	2530.99	29.65	6349.85
**Heart Chambers**					
Left Atrium D2 cc	678.94	66.51	1552.71	25.12	4433.14
Left Atrium D45%	77.11	32.67	536.6	12.3	1403.36
Right Atrium D2 cc	419.61	41.52	1235.79	14.02	4909.75
Right Atrium D45%	37.6	18.02	120.8	6.01	2124.36
Left Ventricle D2 cc	368.04	42.06	1041.32	11.39	4488.94
Left Ventricle D45%	32.97	13.75	85.65	4.69	1910.28
Right Ventricle D2 cc	234.58	35.5	900.28	11.28	1903.36
Right Ventricle D45%	25.59	11.09	83.05	4.62	1530.24
**Heart Valves**					
Aortic valve D2 cc	179.01	38.5	879.52	12.26	2308.88
Aortic valve Mean Dose	87.1	28.64	500.68	13.32	1579.56
Pulmonary valve D2 cc	108.12	32.23	498.07	13.31	2647.47
Pulmonary valve Mean Dose	118.84	31.94	383.09	12.15	1845.57
Mitral valve D0.1 cc	83.56	28.1	474.67	9.48	2446.96
Mitral valve Mean Dose	54.41	24.64	244.7	7.7	2245.14
Tricuspid valve D0.1 cc	36.39	15.1	332.02	6.43	1622.2
Tricuspid valve Mean Dose	27.68	12.25	112.23	4.08	1471.19
**Coronary Arteries**					
Left Main Coronary D0.1 cc	120.13	38.78	560.59	18.34	2117.29
Left Main Coronary Mean Dose	113.89	36.03	477.5	16.7	1783.02
LAD D0.1 cc	293.01	46.09	1181.31	18.11	4953.49
LAD Mean Dose	103.58	25.44	472.11	7.45	3739.5
Left Circumflex D0.1 cc	293.1	40.59	909.36	16.04	2229.17
Left Circumflex Mean Dose	129.48	35.11	580.32	7.7	1791.6
Right Coronary D0.1 cc	63.11	23.76	756.12	9.85	2237.97
Right Coronary Mean Dose	48.61	22.26	366	7.87	1625.13

**Table 3:** Univariate Cox regression.

Dose Constraint	*p*-Value
Left Circumflex D0.1 cc	0.92
Left Circumflex Mean Dose	0.99
LAD D0.1 cc	0.42
LAD Mean Dose	0.8
Left Main Coronary D0.1 cc	0.85
Left Main Coronary Mean Dose	0.76
Pulmonary Artery D10 cc	0.85
Pulmonary Artery Dmax	0.44
Right Coronary D0.1 cc	0.27
Right Coronary Mean Dose	0.14
Aortic valve D2 cc	0.29
Aortic valve Mean Dose	0.24
Left Atrium D2 cc	0.53
Left Atrium D45%	0.43
Right Atrium D2 cc	0.21
Right Atrium D45%	0.021
Heart/Pericardium D15 cc	0.92
Heart/Pericardium Dmax	0.57
Heart D10 cc	0.44
Heart D45%	0.46
Mitral valve D0.1 cc	0.77
Mitral valve Mean Dose	0.53
PTV Volume covered by 100%	0.65
PTV Volume covered by 90%	0.74
Pulmonary Artery D10 cc	0.93
Pulmonary Artery Dmax	0.85
Tricuspid valve D0.1 cc	0.56
Tricuspid valve Mean Dose	0.86
Superior Vena Cava D2 cc	0.77
Superior Vena Cava Dmax	0.92
Left Ventricle D2 cc	0.34
Left Ventricle D45%	0.97
Right Ventricle D2 cc	0.44
Right Ventricle D45%	0.012

**Table 4:** Multivariate Cox regression.

	Non-Cancer Associated Survival		Overall Survival	
	HR (95% CI for HR)	*p*-Value	HR (95% CI for HR)	*p*-Value
Gender (Female)			0.50 (0.27–0.91)	0.02
KPS (<80)	4.1 (1.8–8.8)	<0.001	2.5 (1.4–4.6)	0.003
Prior lung cancer	0.2 (0.08–0.7)	0.011	0.75 (0.37–1.5)	0.42
History of diabetes			2.6 (1.4–4.6)	0.002
Heart disease	1.2 (0.57–2.7)	0.58	0.7 (0.38–1.3)	0.27
PTV			1.0 (0.99–1.02)	0.14
Bronchus D4 cc	2.1 (0.8–5.2)	0.1	2.2 (0.93–5.1)	0.074
Trachea D4 cc	3.8 (1.0–11.1)	0.015	2.7 (1.0–7.3)	0.051
Right Atria D45%	8.0 (1.0–62.5)	0.048	7.4 (1.2–45.7)	0.029
Right Ventricle D45%	0.35 (0.04–3.3)	0.36	0.31 (0.05–2.1)	0.22

## Data Availability

Drs. Farrugia, Malhotra, Singh had full access to all the data in the study and take responsibility for the integrity of the data and the accuracy of the data analysis. The data underlying this article cannot be shared publicly for the privacy of individuals that participated in the study. The data are available from the corresponding authors upon reasonable request.

## Supplementary Materials

The following supporting information can be downloaded at: https://www.mdpi.com/article/10.3390/cancers14061391/s1, Table S1: Heart disease by specific location; Table S2: Univariate Cox regression for overall survival; Table S3: Multivariate analysis with Ultracentral tumor location.
