# Supplementary material for: Right Atrial Dose Is Associated with Worse Outcome in Patients Undergoing Definitive Stereotactic Body Radiation Therapy for Central Lung Tumors
Source: Cancers (Basel). 2022 Mar 9;14(6):1391. doi: 10.3390/cancers14061391 (PMC8945864; doi:10.3390/cancers14061391)
Supplement: Supplementary file 1 [file cancers-14-01391-s001.zip › cancers-1582289-supplementary.pdf]

Supplemental Table S1. Heart disease by specific locations.

Heart disease (*n* = 34, 40.5%)
CHF (*n* = 15, 44.1%)
CAD (*n* = 30, 88.2%)
MI (*n* = 13, 38.2%)
AICD/Pacer (*n* = 6, 17.6%)

Supplemental Table S2. Univariate Cox regression for overall survival

	HR (95% CI for HR)	*p*.value
A_Ascending_Arot_D10cc	1 (1-1)	0.23
A_Ascending_Arot_Maximum_Dose	1 (1-1)	0.14
A_Circumflex_Left_D0.1cc	1 (1-1)	0.9
A_Circumflex_Left_Mean_Dose	1 (1-1)	0.9
A_Descending_Aor_D10cc	1 (1-1)	0.71
A_Descending_Aor_Maximum_Dose	1 (1-1)	0.8
A_LAD_D0.1cc	1 (1-1)	0.67
A_LAD_Mean_Dose	1 (1-1)	0.47
A_LM_Coronary_D0.1cc	1 (1-1)	0.61
A_LM_Coronary_Mean_Dose	1 (1-1)	0.56
A_Pulmonary_D10cc	1 (1-1)	0.98
A_Pulmonary_Maximum_Dose	1 (1-1)	0.59
A_R_Coronary_D0.1cc	1 (1-1)	0.38
A_R_Coronary_Mean_Dose	1 (1-1)	0.1
Aortic_valve_D2cc	1 (1-1)	0.36
Aortic_valve_Mean_Dose	1 (1-1)	0.18
Atrium_Left_D2cc	1 (1-1)	0.2
Atrium_Left_D45.	1 (1-1)	0.15
Atrium_Right_D2cc	1 (1-1)	0.32
Atrium_Right_D45.	1 (1-1)	**0.011**
Heart.Pericardium_D15cc	1 (1-1)	0.81
Heart_Whole_D10cc	1 (1-1)	1
Heart_Whole_D45.	1 (1-1)	0.25
Heart_Whole_Maximum_Dose	1 (1-1)	0.43
Mitral_valve_D0.1cc	1 (1-1)	0.52
Mitral_valve_Mean_Dose	1 (1-1)	0.92
PTV_Volume_covered_by_100.Rx_Dose	0.94 (0.73-1.2)	0.61
PTV_Volume_covered_by_90.Rx_Dose	1 (0.72-1.5)	0.86
Tricuspid_valve_D0.1cc	1 (1-1)	0.47
Tricuspid_valve_Mean_Dose	1 (1-1)	0.78
V_Superior_VenaC_D2cc	1 (1-1)	0.94
V_Superior_VenaC_Maximum_Dose	1 (1-1)	0.83
Ventricle_Left_D2cc	1 (1-1)	0.51
Ventricle_Left_D45.	1 (1-1)	0.99
Ventricle_Right_D2cc	1 (1-1)	0.48
Ventricle_Right_D45.	1 (1-1)	**0.016**

Supplemental Table S3. Multivariate analysis with Ultracentral tumor location

	**Non-cancer associated survival**		**Overall Survival**	
	HR (95% CI for HR)	*p*-value	HR (95% CI for HR)	*p*-value
Gender (Female)			0.46 (0.26-0.82)	0.01
KPS (<80)	3.8 (1.8-8.2)	<0.001	2.2 (1.2-4.0)	0.009
Prior lung cancer	0.2 (0.07-0.6)	0.006	0.51 (0.25-1.1)	0.07
History of diabetes			2.2 (1.2-3.9)	0.007
PTV			1.0 (0.99-1.01)	0.45
Ultracentral	1.8 (0.9-3.9)	0.12	2.4 (1.25-4.6)	0.009
Right Atria D45%	6.6 (0.9-48.2)	0.062	8.2 (1.3-51.5)	0.024
Right Ventricle D45%	0.4 (0.05-3.6)	0.37	0.26 (0.04-1.9)	0.18
